# Calprotectin, a new biomarker for diagnosis of acute respiratory infections

**DOI:** 10.1038/s41598-020-61094-z

**Published:** 2020-03-06

**Authors:** Aleksandra Havelka, Kristina Sejersen, Per Venge, Karlis Pauksens, Anders Larsson

**Affiliations:** 1 Department of Molecular Medicine and Surgery, Karolinska Institute, Stockholm, Sweden; 2 Gentian Diagnostics AB, Stockholm, Sweden; 3 Department of Medical Sciences, Clinical Chemistry, Uppsala University, Uppsala, Sweden; 4 Department of Medical Sciences, Infectious Disease, Uppsala University, Uppsala, Sweden

**Keywords:** Bacteria, Infectious-disease diagnostics, Diagnostic markers, Infectious diseases, Bacterial infection, Viral infection

## Abstract

Respiratory tract infections require early diagnosis and adequate treatment. With the antibiotic overuse and increment in antibiotic resistance there is an increased need to accurately distinguish between bacterial and viral infections. We investigated the diagnostic performance of calprotectin in respiratory tract infections and compared it with the performance of heparin binding protein (HBP) and procalcitonin (PCT). Biomarkers were analyzed in patients with viral respiratory infections and patients with bacterial pneumonia, mycoplasma pneumonia and streptococcal tonsillitis (n = 135). Results were compared with values obtained from 144 healthy controls. All biomarkers were elevated in bacterial and viral infections compared to healthy controls. Calprotectin was significantly increased in patients with bacterial infections; bacterial pneumonia, mycoplasma pneumonia and streptococcal tonsillitis compared with viral infections. PCT was significantly elevated in patients with bacterial pneumonia compared to viral infections but not in streptococcal tonsillitis or mycoplasma caused infections. HBP was not able to distinguish between bacterial and viral causes of infections. The overall clinical performance of calprotectin in the distinction between bacterial and viral respiratory infections, including mycoplasma was greater than performance of PCT and HBP. Rapid determination of calprotectin may improve the management of respiratory tract infections and allow more precise diagnosis and selective use of antibiotics.

## Introduction

Acute respiratory infections are common worldwide, and in many countries constitute a major cause of mortality and morbidity^1^. The underlying pathogenetic agents behind acute respiratory infections vary geographically^2^, but the clinical importance of early diagnosis is universal. Two major reasons for the importance of early diagnosis are firstly to minimize time from start of symptom until initiation of proper medical therapy to reduce risks of protracted infection, sepsis/mortality, and late sequelae^3^. A second reason is to avoid improper use of antibiotics in cases where this is not indicated, emphasized by an increased universal problem with antibiotic resistance^4^. Within a few years after the discovery of penicillin, resistance to penicillin was observed and today antibiotic resistance has become a substantial clinical problem^5^. Bacterial infections have thus again reoccurred as a major threat to our health. To preserve the effectiveness of antibiotics it is important to use antibiotics more selectively and avoid unnecessary use. Biomarkers, which in early stage of an infection can distinguish between bacterial and viral infection could lead to a more selective use of antibiotics^3^.

Since isolation of the disease-causing microorganism is usually too time consuming to be useful for early diagnosis, other biomarkers are used clinically to distinguish viral infections not requiring antibiotic treatment from bacterial infections in early phases of the infection. Such biomarkers usually include a combination of white blood cell count, neutrophil count, C-reactive protein, and less frequently procalcitonin (PCT), heparin binding protein (HBP) or calprotectin^6,7^.

Procalcitonin (PCT) is a precursor to the hormone calcitonin, the latter being involved in calcium homeostasis. Together with CRP, white blood cells and neutrophils, procalcitonin is one of the markers most widely used to distinguish between bacterial and viral infections^8^. Heparin binding protein (HBP) and calprotectin are expressed mainly in neutrophil granulocytes^9,10^. HBP is stored in azurophil granules while calprotectin is stored in the cytosol^11,12^. Both are used as markers of neutrophil activation. Calprotectin is one of the most abundant proteins in the cytosol of neutrophil granulocytes, where it accounts for 40–50% of the total protein content. Calprotectin is released upon activation and turnover of neutrophils and is recognized as an important marker for neutrophil mediated inflammation^13^.

Bacterial and viral infections cause an acute phase response which will counterattack the infection and reduce the damage. The infection leads to an activation of the innate immune system which is an early defense mechanism. The major functions of the innate immunity include the recruitment of immune cells to the site of infection, activation of the complement cascade and activation of white blood cells to eliminate the microorganisms. This is a rapid process and the neutrophil is the first cell to reach the affected location^14^. Neutrophil activation markers will be released from the neutrophils upon activation. These activation markers are stored in granulae or cytoplasm and can be rapidly released from the neutrophils^15^. As there is no need for de novo synthesis, a neutrophil marker should be an earlier marker of neutrophil activation caused by bacterial infections in comparison with formation of new white blood cells or synthesis of proteins.

The aim of this study was to investigate the performance of calprotectin as a marker for bacterial infections and its possibility to distinguish between bacterial, mycoplasma, and viral respiratory tract infections.

## Results

### Patient characterization

Serum and plasma samples were collected from patients with fever of >38 °C and symptoms of respiratory infections and from asymptomatic healthy subjects.

Infected patients were classified as having a bacterial, mycoplasma or viral cause of their disease. The primary study group included patients with confirmed etiology of respiratory infections. The study group consisted of 279 subjects (144 asymptomatic healthy controls, 71 with bacterial infections, 24 with mycoplasma infections and 40 with viral infections) (Table 1).

**Table 1:** Patients with signs and symptoms of acute respiratory infections with confirmed etiology.

Type of infection	No. of patients with confirmed etiology	Age (median and range) (% male)
Viral	40	48.0 ± 20.1 (49%)
Bacterial pneumonia	34	64.4 ± 15.8 (47%)
Mycoplasma pneumonia	24	40 ± 15.5 (42%)
Streptococcal tonsillitis	37	34.1 ± 10.6 (32%)

Viral respiratory disease: Influenza A (n = 19), RSV (n = 5), Influenza B (n = 4), CMV (n = 2), Rhinovirus (n = 2), VZV (n = 2), Coronavirus (n = 1), Parainfluenza (n = 1), Dengue (n = 1), HSV (n = 1).

Bacterial pneumonia: Pneumococcus (Streptococcus pneumoniae) (n = 23) and H. influenzae (n = 11).

### Diagnostic performances of calprotectin, procalcitonin and heparin binding protein (HBP)

**Figure 1:**
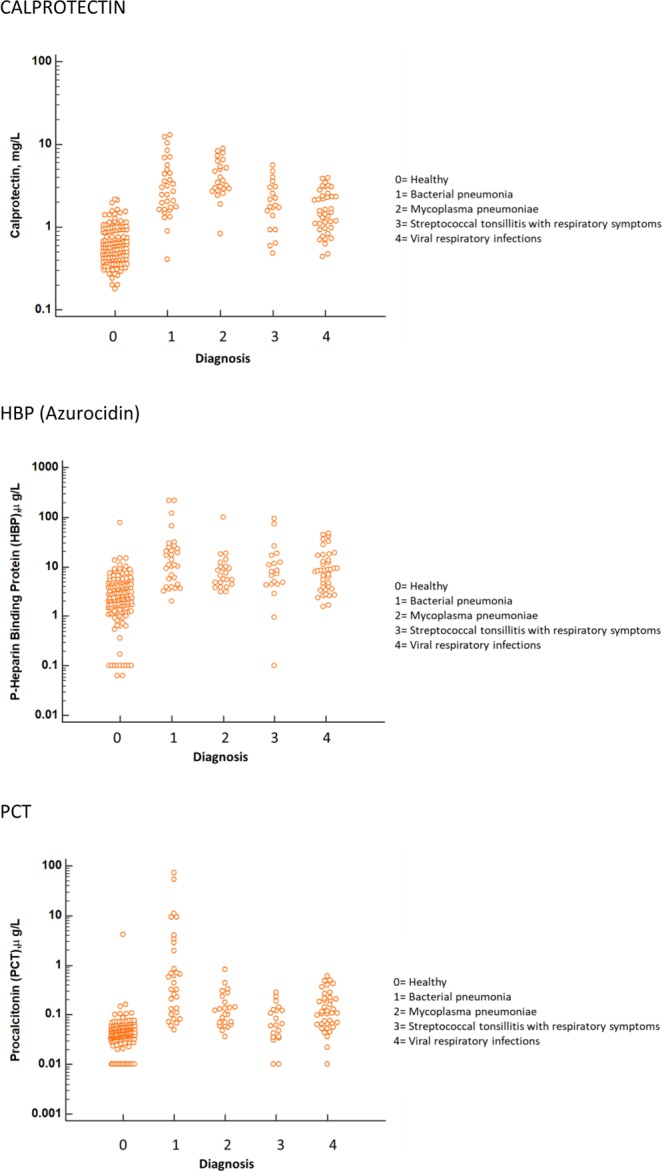
Biomarker concentrations in the different diagnostic groups, as indicated by the numbers. Top: Calprotectin, Middle: HBP (Azurocidin), Bottom: Procalcitonin. For all panels, only results with verified etiologies are presented.

The distribution of Calprotectin, Procalcitonin, and HBP concentrations in patients with respiratory tract infections, separated by verified clinical diagnosis is presented in Fig. 1.

Compared to healthy subjects, all three biomarkers were significantly elevated in all diagnostic groups (***P < 0.001). Calprotectin levels were significantly higher in patients with bacterial pneumonia, mycoplasma pneumonia (zzz, p < 0.001) and Streptococcal tonsillitis (z, p < 0.05) compared to levels in patients with viral infections.

Concentration of PCT in bacterial pneumonia was significantly higher (zzz, p < 0.001) than the concentration in the viral group, whereas the levels in Streptococcal tonsillitis and in Mycoplasma pneumoniae were not significantly different compared to levels in patients with viral infections. HBP concentration was not significantly different in mycoplasma and bacterial infections compared to viral infections. The results are summarized in Table 2.

**Table 2:** Concentrations of the studied biomarkers and the possibility to differentiate between healthy population and infected patients (*) as well as between patients with bacterial and viral infections (z).

Group	Calprotectin (mg/L) Median (IQ range)	HBP (μg/L) Median (IQ range)	PCT (µg/L) Median (IQ range)
Healthy	0.61 (0.43–0.92) n = 144	2.24 (1.40–4.28) n = 144	0.042 (0.034–0.057) n = 144
Viral respiratory infections	1.47 (0.98–2.35) n = 40***	8.24 (3.69–15.3) n = 40***	0.109 (0.065–0.213) n = 39***
Bacterial pneumonia	2.69 (1.66–4.8) n = 31***^, zzz^	11.0 (4.71–24.1) n = 31***	0.443 (0.117–2.64) n = 31***^, zzz^
Mycoplasma pneumonia	3.52 (2.84–5.81) n = 24***^, zzz^	6.59 (4.47–10.1) n = 23***	0.112 (0.067–0.207) n = 24***
Streptococcal tonsillitis	2.14 (1.37–3.22) n = 37***^, z^	7.59 (4.37–16.6) n = 37***	0.085 (0.041–0.131) n = 37***

***p < 0.001, bacterial, viral or mycoplasma infections vs healthy population.

^zzz^p < 0.001, ^z^p < 0.05, bacterial or mycoplasma infections vs viral infections.

**Figure 2:**
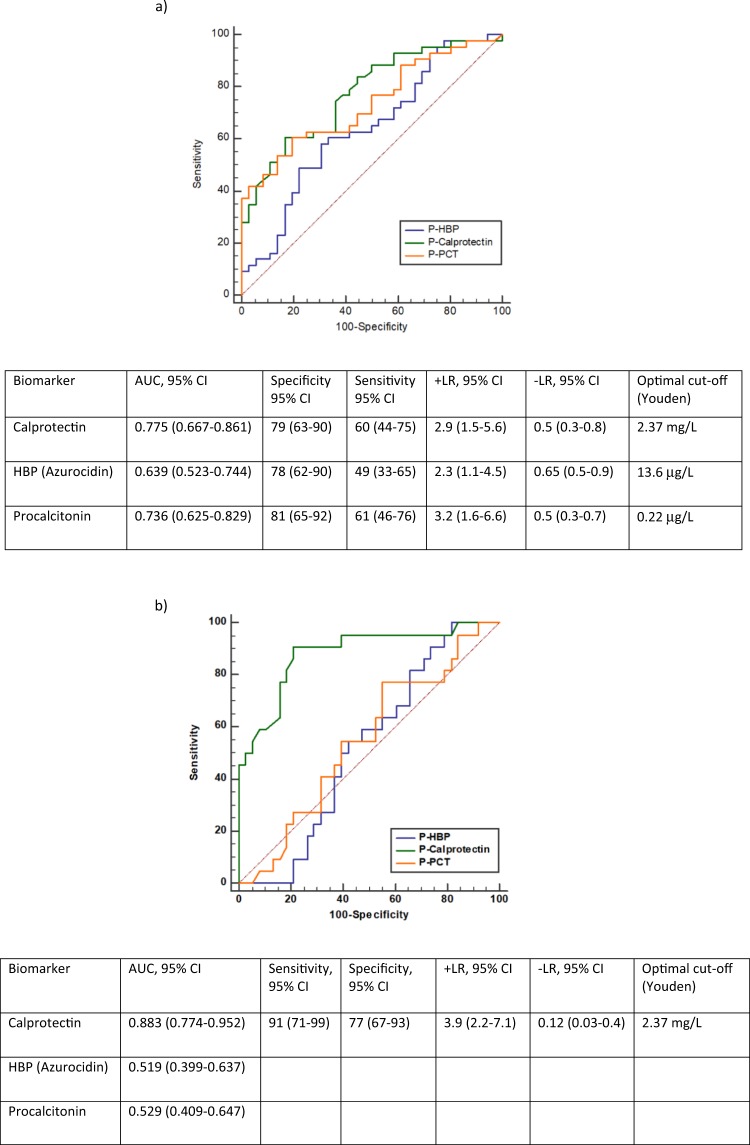
(**a**) Receiver operating characteristic curves, AUC, Sensitivity and Specificity of Calprotectin, HBP and Procalcitonin in the discrimination between patients with bacterial pneumonia and viral respiratory infections. (**b**) Receiver operating characteristic curves, AUC, Sensitivity and Specificity of Calprotectin, HBP and Procalcitonin in the discrimination between patients with mycoplasma pneumonia and viral respiratory infections.

Figure 2 shows ROC curves and diagnostic performance of all three biomarkers in differentiation between bacterial pneumonia (Fig. 2a), mycoplasma pneumonia (Fig. 2b) and viral infections.

Performance of Calprotectin in differentiation between bacterial, mycoplasma and viral infections was higher than performance of HBP and PCT and is most prominent in the differentiation between mycoplasma- and viral infections.

In Table 3, the AUROC results for the three biomarkers are summarized for the distinction between diagnosis of bacterial pneumonia, mycoplasma pneumonia and viral infections.

**Table 3:** The AUROC results for the three biomarkers are summarized for distinction between bacterial and mycoplasma pneumonia vs respiratory viral infections.

Diagnosis	AUROC (95% CI)		
	Calprotectin	HBP	PCT
Bacterial pneumonia	0.76 (0.67–0.86)	0.64 (0.52–0.74)*	0.74 (0.63–0.82)
Mycoplasma pneumonia	0.88 (0.77–0.95)	0.52 (0.40–0.64)***	0.53 (0.41–0.65)***

***p<0.001, *p<0.05, calprotectin vs other biomarkers in differentiation between bacterial, mycoplasma and viral infections.

## Discussion

The aim of the current study was to evaluate the performance of calprotectin, a neutrophil activation marker for the diagnosis of acute respiratory infection and the differentiation between bacterial and viral infections. Performance of calprotectin was compared to performance of heparin binding protein (HBP) which also is produced in neutrophils but stored in the azurophil granules within the cells and procalcitonin (PCT) which is widely used as a marker for bacterial infections. We found significantly higher plasma calprotectin values in patients with bacterial respiratory infections (pneumonia, tonsillitis, or mycoplasma) than in patients with viral respiratory infections or healthy controls. The AUC for calprotectin and PCT were higher than the AUC for HBP when used for diagnosis of bacterial pneumonia, mycoplasma pneumonia and streptococcal tonsillitis (Fig. 2, Table 3). Furthermore, the AUC for calprotectin was significantly higher than that for both PCT and HBP in discriminating mycoplasma infections from viral infections. These data support clinical use of calprotectin in diagnosis of respiratory infections and indicate greater performance of calprotectin compared to HBP and PCT in distinguishing bacterial and mycoplasma respiratory infections from viral infections. Our findings are supported by observations from other studies about the limited clinical utility of procalcitonin in diagnosis of bacterial pneumonia^16^.

The response of calprotectin to bacterial respiratory infections is well in line with previous reports demonstrating increase in calprotectin in sepsis^17–20^ as well as other inflammatory conditions with neutrophil activation such as appendicitis, synovitis and prosthetic joint infections^21–23^.

The performance of CRP and White blood cell counts (WBC) was not evaluated in this study since they were used in the clinical judgment of the diagnosis and a bias toward these two biomarkers hindered an accurate evaluation of their diagnostic performance. In earlier publications it has been shown that CRP is a powerful tool for ruling out patients with bacterial infections. In viral infections, however, the considerable overlap with bacterial infections, indicated that CRP is less useful in this distinction^3,24–27^.

This study has certain limitations. It is a single-center study with limited number of patients in different diagnostic groups. However, all diagnoses are well defined and confirmed by X-ray, blood/tissue cultures and/or PCR analysis. Our data suggest that measuring plasma calprotectin in patients with suspected respiratory infections is feasible, with possibility for fast test results. The results of this study may aid in the design of the future studies.

In the present study we used plasma samples for the determination of calprotectin and HBP as the coagulation process necessary for preparing serum samples causes activation of neutrophils and could thus lead to elevated levels of calprotectin and HBP when analyzed in serum. PCT should not be affected by the coagulation process and was thus analyzed in serum samples. The HBP assay is a microtiter-based ELISA which is associated with very long test turnaround times as the test format requires collection of a number of samples prior to testing. The available commercial procalcitonin assays are either microtiter based or developed for specific assay platforms and these platforms are not available in all laboratories. This means that the samples may have to be sent to another laboratory for testing which also increases the time until the test results are available. The calprotectin assay used was a particle enhanced turbidimetric assay (PETIA) with an assay time of 10 min. PETIA is a technology that can be applied on routine chemistry analyzers available at most hospital laboratories allowing short test turnaround times. In comparison with ELISA it is designed for measuring single samples, and thus, shortens the analysis times significantly. This is important as bacterial infections are acute condition that requires rapid test results. In conclusion, calprotectin and procalcitonin performed better than HPB in distinguishing between pneumonia and tonsillitis caused by bacteria and viral infections. Furthermore, calprotectin was superior to HBP and procalcitonin in distinguishing between respiratory infections caused by mycoplasma and viruses in primary care and infectious disease unit patients. Our results indicate that calprotectin is altogether more sensitive than HBP and procalcitonin in distinguishing bacterial and mycoplasma from viral cause of respiratory infections.

## Methods

### Patients

The inclusion criteria for patients in the study were fever of >38 °C and signs and symptoms of respiratory infection. The inclusion and diagnosis of the patients have been described in detail previously^24^.

The exclusion criterion was known chronic viral infection, such as human immunodeficiency virus infection or hepatitis. In addition, children age <18 years and patients who could not give informed consent were excluded from this study. The patients were admitted to the infectious disease department at the University Hospital in Uppsala or to a primary care unit in Uppsala. A blood sample was drawn before the start of antibiotic treatment. The study was approved by the Uppsala Regional Ethics Committee, Uppsala, Sweden. All parts of the study were performed in accordance with the ethical approval^24^ and Swedish and European regulations. All participants gave informed consent prior to inclusion in the study.

In this study 144 healthy individuals and 135 patients with a confirmed etiology of their acute respiratory infection were included. Of these patients, 95 had a bacterial infection and 40 had a viral infection. The 144 healthy controls consisted of 57 males (age 41.3 ± 12.7 years, mean ± SD) and 87 females (age 45.0 ± 12.8 years, mean ± SD). The age and gender distribution of the confirmed infections are shown in Table 1.

In the patient group, clinical findings and assessment were documented, including white blood cell counts and CRP levels, and verified with objective tests used in the routine diagnostics. In the pneumonia group, the diagnosis was verified with a positive chest X-ray and supported by positive culture or PCR test from the lower respiratory tract samples. The diagnosis of respiratory tract infection with viruses, for example, influenza A/B and atypical pneumonia, such as mycoplasma pneumonia, was supported by PCR testing of samples from the respiratory tract. Viral infections, such as dengue fever, Epstein-Barr virus, or cytomegalovirus infection, were supported by IgG/IgM serology results. The diagnosis of bacterial infections was supported by cultures from the respiratory tract, when appropriate. Tonsillitis was diagnosed by a rapid test for group A Streptococcus and supported by positive culture.

### Biomarkers

Calprotectin was measured in plasma samples with turbidimetric method (Gentian AS, Norway) on Mindray BS 280 Instrument. Heparin binding protein (HBP, Azurocidin) was analyzed by a commercially available ELISA (HK352, Hycult Biotech, Uden, The Netherlands). Procalcitonin was analyzed using a sandwich ELISA (EHPCT) from Thermo Fisher Scientific (Frederick, MD, USA). Calprotectin and HBP were analyzed in EDTA plasma while procalcitonin was analyzed in serum. The plasma and serum tubes were collected at the same time.

### Statistics

The data of groups are expressed as medians and interquartile range (IQ) wherever appropriate. Comparison between groups was performed by the non-parametric Mann-Whitney-U test. The clinical performances of the biomarkers were tested by receiver operating characteristics (ROC) giving the area under the curve (AUC). The AUCs were compared by c-statistics. Youden index was calculated for the estimation of the optimal cut-off and used to define sensitivity, specificity, positive and negative likelihoods. All analyses were performed by the statistical program MedCalc Statistical Software (Ostend, Belgium).
